# Prevalence of bruxism in patients affected by epilepsy: a systematic review and meta-analysis

**DOI:** 10.2340/aos.v84.42959

**Published:** 2025-04-02

**Authors:** Giuseppe Minervini, Rocco Franco, Marco Di Blasio, Mirko Martelli, Marco Gargari, Patrizio Bollero, Marco Cicciù

**Affiliations:** a Saveetha Dental College and Hospitals, Saveetha Institute of Medical and Technical Sciences (SIMATS), Saveetha University, Chennai, India; b Multidisciplinary Department of Medical-Surgical and Dental Specialties, University of Campania 'Luigi Vanvitelli', Naples, Italy; c Department of Biomedicine and Prevention, University of Rome ‘Tor Vergata’, Rome, Italy; d Department of Biomedical Surgical and Dental Sciences, University of Milan, Milan, Italy; e Department of Clinical Sciences and Translational Medicine, University of Rome ‘Tor Vergata’, Rome, Italy; f Department of System Medicine, University of Rome Tor Vergata, Rome, Italy; g Department of Biomedical and Surgical and Biomedical Sciences, Catania University, Catania, Italy

**Keywords:** Temporomandibular disorders, TMD, bruxism, epilepsy

## Abstract

**Background:**

Bruxism, defined by the involuntary grinding or clenching of teeth, and epilepsy, a neurological ailment marked by recurring seizures, are both common conditions that can significantly affect persons’ quality of life. Although numerous studies have investigated the relationship between bruxism and epilepsy, the current evidence is ambiguous. This systematic review seeks to consolidate existing information to elucidate the association between bruxism and epilepsy.

**Methods:**

In accordance with the Preferred Reporting Items for Systematic Reviews and Meta-Analyses (PRISMA) guideline, a systematic search was performed across electronic databases, including PubMed, Lilacs, and Web of Science. The search encompassed all pertinent publications published until September 2021. The inclusion criteria were established to encompass observational studies (cohort, case-control, cross-sectional) that investigated the relationship between bruxism and epilepsy in human populations.

**Results:**

The preliminary search produced a total of 142 articles. After a rigorous screening process, 3 studies were declared appropriate for inclusion in the systematic review. The research varied in design, sample size, and methodology, with some studying the prevalence of bruxism in epileptic patients, while others studied the prevalence of epilepsy in individuals with bruxism. Heterogeneity among the research was handled using proper statistical analyses.

**Conclusion:**

The findings from the included studies suggested a probable link between bruxism and epilepsy. However, the evidence was uneven and equivocal, with some research indicating a favourable correlation, while others showed no meaningful relationship. Methodological restrictions, such as changes in diagnostic criteria and data collection procedures, could contribute to the observed inconsistencies. In addition, the possible influence of confounding factors, such as medication use and comorbidities, should be addressed in interpreting the data.

## Introduction

Bruxism and epilepsy are two distinct neurological disorders that have garnered significant attention in the medical community due to their multifaceted nature and potential overlap in clinical manifestations [1, 2]. Bruxism is a behavioural trait characterised by the involuntary grinding or clenching of teeth, often during sleep or periods of heightened stress, resulting in tooth wear and possible tissue damage. Epilepsy on the other hand is a chronic neurological condition characterised by recurrent, unprovoked seizures, resulting from abnormal electrical activity in the brain. Despite being seemingly unrelated conditions, recent research has indicated that there may be an intriguing connection between these two disorders, possibly sharing common underlying mechanisms and clinical implications [1, 2].

Sleep bruxism is a masticatory muscle activity during sleep that is characterised as rhythmic (phasic) or non-rhythmic (tonic), and is not a movement disorder or a sleep disorder in otherwise healthy individuals. Awake bruxism on the other hand is a masticatory muscle activity during wakeful-ness that is characterised by repetitive or sustained tooth contact and/or by bracing or thrusting of the mandible and is not a movement disorder in otherwise healthy individuals [3–7]. In recent decades, the prevalence of bruxism and epilepsy has been steadily increasing, raising concerns about their impact on public health. Bruxism is considered a multifactorial condition with both peripheral and central origins, involving complex interactions between genetic, psychological, and environmental factors. Likewise, epilepsy represents a heterogeneous group of disorders with various aetiologies, affecting individuals of all ages and significantly impairing their quality of life [3–7]. Although seemingly divergent in their nature, researchers have begun to explore the potential link between these two neurological conditions, urging for further investigation into their shared mechanisms and clinical consequences.

A noteworthy area of research linking bruxism and epilepsy is the concept of shared neural pathways and neurotransmitter systems. The central nervous system regulates both motor control, which is involved in bruxism, and seizure generation, which characterises epilepsy. A growing body of evidence suggests that alterations in the balance of neurotransmitters, such as dopamine, serotonin, and gamma-aminobutyric acid (GABA), may play a crucial role in the manifestation of both disorders. In addition, dysregulation of the autonomic nervous system, which has been implicated in bruxism, may also influence seizure susceptibility in epilepsy [8–12].

Moreover, studies have hinted at a possible bidirectional relationship between bruxism and epilepsy. It has been reported that individuals with epilepsy are more likely to experience sleep disturbances, including bruxism episodes, potentially caused by seizure-related disruptions in the sleep-wake cycle. Conversely, chronic bruxism may exacerbate seizure activity by disrupting normal brain function and contributing to increased neuronal excitability [13].

Despite the emerging evidence indicating a potential connection, the association between bruxism and epilepsy remains complex and not fully understood. As such, it is imperative to undertake a review of the current literature to consolidate existing findings and identify knowledge gaps that warrant further exploration. By better understanding the underlying mechanisms linking these two disorders, we may pave the way for improved diagnostic and therapeutic strategies that encompass both conditions, ultimately enhancing patient outcomes and overall quality of life.

In this review, we aim to synthesise the existing research on bruxism and epilepsy, encompassing their epidemiology, pathophysiology, clinical features, and potential shared mechanisms [14]. In addition, we will critically assess the available evidence regarding their bidirectional relationship, exploring the implications for diagnostic practices and therapeutic interventions. By elucidating the intricate interplay between bruxism and epilepsy, we hope to foster a broader understanding of these neurological conditions and inspire future investigations into novel therapeutic avenues for patients affected by their co-occurrence [15].

Furthermore, investigating the relationship between bruxism and epilepsy can potentially shed light on the broader understanding of the pathophysiology of both conditions. As we delve deeper into the intricate mechanisms underlying their co-occurrence, we may uncover shared genetic factors or biological pathways that could explain their association. This, in turn, could open up new avenues for targeted therapeutic interventions that address the common mechanisms, providing relief to patients experiencing both disorders simultaneously [16].

Epidemiological studies focussed on the prevalence and incidence rates of bruxism in individuals with epilepsy and vice versa will be instrumental in determining the strength of the association between these two conditions. Longitudinal studies examining the temporal relationship between the onset or exacerbation of bruxism and epilepsy seizures could offer insights into causality and directionality.

Moreover, the impact of bruxism on epilepsy management and vice versa necessitates thorough exploration [17]. For individuals with epilepsy, sleep disturbances due to bruxism episodes may exacerbate seizure frequency and intensity. This emphasises the importance of early identification and management of bruxism in patients with epilepsy, potentially leading to better seizure control and improved overall well-being. On the other hand, the impact of epileptic seizures on bruxism warrants investigation, as uncontrolled seizures might aggravate the severity of bruxism, potentially leading to or exacerbating dental complications and temporomandibular joint disorders.

A multidisciplinary approach involving neurologists, dentists, sleep specialists, and other healthcare professionals will be essential to comprehensively understand the complex relationship between bruxism and epilepsy. Collaboration among these disciplines can facilitate a holistic assessment of patients, encompassing both neurological and dental evaluations, enabling a more accurate diagnosis and targeted management plan [18].

With the advent of wearable devices, advanced imaging techniques, and non-invasive monitoring tools, there is an opportunity to gain a more in-depth understanding of the physiological changes associated with both bruxism and epilepsy. Capturing real-time data on muscle activity, brain electrical patterns, and autonomic responses during episodes of bruxism and seizures could provide valuable insights into the underlying mechanisms and potential triggers.

In conclusion, the exploration of the connection between bruxism and epilepsy represents an exciting and promising area of research in the fields of neurology and dentistry. As we continue to unravel the shared pathways, neural mechanisms, and clinical implications of these two neurological disorders, we move closer to identifying novel therapeutic strategies and preventive measures that could improve the quality of life for individuals affected by either condition or their co-occurrence [19, 20]. By fostering collaborative research efforts and leveraging cutting-edge technologies, we can aspire to better understand and address the challenges posed by bruxism and epilepsy, ultimately leading to improved patient care and outcomes.

In addition to the biological and neurological aspects, it is imperative to consider the psychosocial dimensions of the relationship between bruxism and epilepsy. Both disorders can profoundly impact an individual’s overall well-being and quality of life, leading to emotional distress, social isolation, and diminished self-esteem.

Living with epilepsy often entails coping with unpredictable seizures and the potential limitations they impose on daily activities. Such challenges can result in increased stress and anxiety, which may exacerbate bruxism in some individuals. Conversely, chronic bruxism can lead to discomfort, pain, and disrupted sleep, which might contribute to emotional distress and potentially affect seizure thresholds in individuals with epilepsy [21].

Furthermore, sleep plays a critical role in both conditions. Sleep disturbances are commonly reported in individuals with epilepsy and can be triggered by seizures or antiepileptic medications. Bruxism episodes occurring during sleep can further disrupt the sleep architecture, leading to a negative impact on daytime functioning, cognitive performance, and seizure control [22–24].

Understanding the psychosocial impact of bruxism and epilepsy, and their potential interaction can help healthcare professionals develop comprehensive treatment plans that address the physical, emotional, and social aspects of these conditions. Integrating psychological support and counselling into the management of patients with bruxism and epilepsy can empower individuals to cope with their conditions more effectively and improve their overall quality of life.

As researchers continue to investigate the relationship between bruxism and epilepsy, it is essential to consider potential confounding factors that may influence the association. Comorbidities, such as anxiety, depression, or other neurological or dental conditions, could contribute to the observed relationship between the two disorders. Therefore, well-designed studies that control for confounding variables are necessary to establish a more definitive and reliable connection [25].

Apart from this association with epilepsy, another good question would be if there is any association between epilepsy and temporomandibular joint dysfunctions (TMDs). Although bruxism and TMDs are two distinct conditions, they could well share common risk factors or pathophysiology. The prevalence of TMDs in patients with epilepsy will be useful in terms of understanding whether these two conditions are indeed potentially comorbid. Therefore, the objective of our systematic review was to determine the prevalence of bruxism and temporomandibular disorders in patients with epilepsy, and to explore associations with the potential impact of antiepileptic drugs (AEDs).

## Materials and methods

### Eligibility criteria

All documents were assessed for eligibility based on the Population, Exposure, Comparator, and Outcomes (PECO) model [26].

P) Participants consisted of human subjects.
E) The Exposure consisted of Epilepsy
C) The Comparison was with not affected by Epilepsy
O) The Outcome consisted of the prevalence of temporomandibular disorders and bruxism in Epilepsy patients.

Only papers providing data at the end of the intervention were included. Exclusion criteria were as follows: (1) patients suffering from other systemic diseases; (2) patients who have fibromyalgia; (3) congenital abnormality or neoplastic conditions in the temporomandibular joint (TMJ) region; (4) patients undergoing drug treatment; (5) studies written in a language different from English; (6) full-text unavailability (i.e. posters and conference abstracts); (7) studies involving animals; (8) review (topical or systematic) articles; and (9) case reports/series.

### Search strategy

We systematically searched Web of Science, PubMed and Lilacs for articles published from the inception until February 2024. We followed the strategy reported in Table 1. Furthermore, we manually searched the references and previous systematic reviews on a similar topic.

**Table 1:** Search strategy.

***PubMed*** * Search: ((TEMPOROMANDIBULAR DISORDERS) OR (BRUXISM)) AND (epilepsy) Filters: English* * ((‘temporomandibular joint disorders’[MeSH Terms] OR (‘temporomandibular’[All Fields] AND ‘joint’[All Fields] AND ‘disorders’[All Fields]) OR ‘temporomandibular joint disorders’[All Fields] OR (‘temporomandibular’[All Fields] AND ‘disorders’[All Fields]) OR ‘temporomandibular disorders’[All Fields] OR (‘bruxism’[MeSH Terms] OR ‘bruxism’[All Fields])) AND (‘epilepsie’ [All Fields] OR ‘epilepsy’[MeSH Terms] OR ‘epilepsy’[All Fields] OR ‘epilepsies’[All Fields] OR ‘epilepsy’[All Fields])) AND (English[Filter])*
***Web of Science*** * (ALL=(bruxism)) AND ALL=(EPILEPSY)*
***Lilacs*** * *BRUXISM [Palavras] or temporomandibular disorders [Palavras] and EPILEPSY [Palavras]

This systematic review was conducted according to the guidance of the Cochrane Handbook for Systematic Reviews of Interventions and the Preferred Reporting Items for Systematic Reviews (PRISMA) guidelines 2020. The systematic review protocol has been registered on the International Prospective Register of Systematic Reviews (PROSPERO) with the number 426037.

### Data extraction

Two reviewers (GM and MC) extracted the data from the included studies using customised data extraction on a Microsoft Excel sheet. In case of disagreement, a consensus was reached through a third reviewer (PB). The following data were extracted: (1) First author; (2) Population; (3) Summary; (4) Significance; (4) Study design; (5) Population characteristics; and (6) Results.

### Quality assessment

For this review, the Risk of Bias in Non-randomized Studies of Interventions (ROBINS-E) tool was utilised to assess the risk of bias in the included studies. The ROBINS-E tool provides a structured framework to evaluate potential biases in non-randomised studies. The assessment process involved independent evaluation by two or more reviewers for each study included in the systematic review. The reviewers were trained in using the ROBINS-E tool and followed the guidelines to evaluate the risk of bias across seven domains: confounding, selection of participants, classification of interventions, deviations from intended interventions, missing data, measurement of outcomes, and selection of reported results. To enhance the objectivity and consistency of the assessments, any discrepancies or disagreements between reviewers were resolved through discussion and consensus. In cases where consensus could not be reached, a third reviewer was involved to make the final determination. The bias assessment using ROBINS-E provided a comprehensive evaluation of the potential biases in the included non-randomised studies. It helps to identify the strengths and limitations of the evidence base, contributing to the overall assessment of the quality and reliability of the findings. By considering the risk of bias, the review authors can make more informed interpretations and draw conclusions based on the available evidence.

### Statistical analysis

We performed the pooled analysis using Review Manager version 5.2.8 (Cochrane Collaboration, Copenhagen, Denmark; 2014). We measured the risk ratio (RR) between the two groups (exposed and not exposed to epilepsy). Heterogeneity among studies was evaluated using the Higgins Index (*I*^2^) and the chi-square test classified as follows: low heterogeneity (< 30%), medium heterogeneity (30–60%), and high heterogeneity (> 60%).

### Grade of strength

We applied the Grading of Recommendations Assessment, Development and Evaluation (GRADE) ranking system to measure the quality of evidence and also to determine the level of certainty for the results of this review.

## Results

### Study characteristics

**Figure 1:**
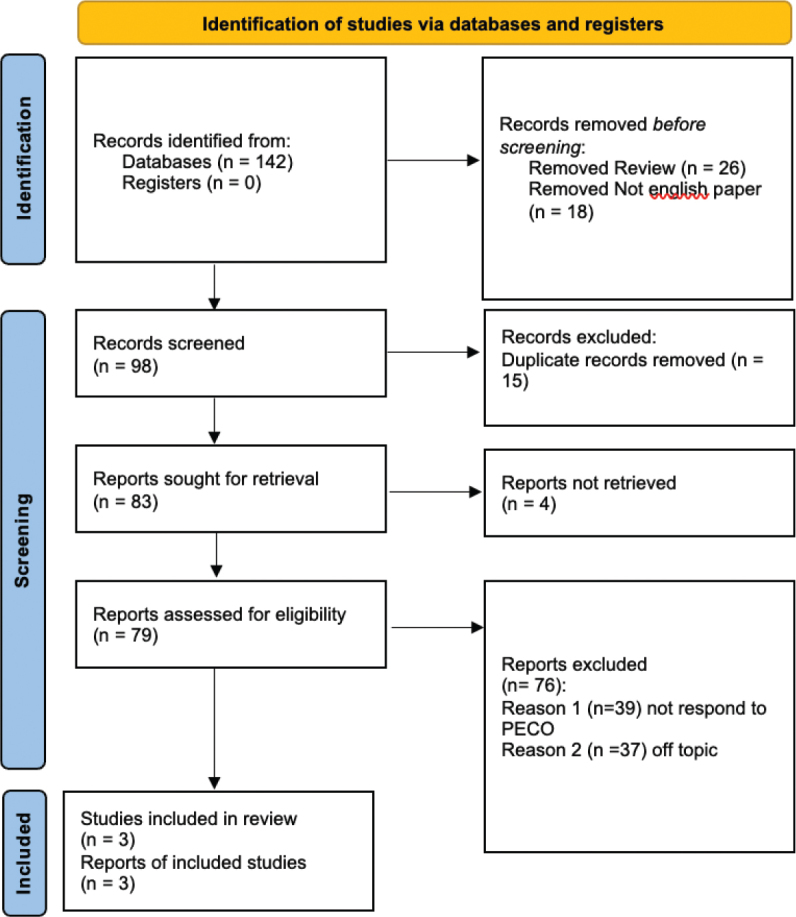
Preferred Reporting Items for Systematic Reviews and Meta-Analyses flowchart.

A total of 142 studies were identified at the end of the research. As illustrated by the PRISMA 2020 flowchart in Figure 1, we chose only 3 studies to draw up the present systematic study. We excluded 44 articles before the screening: 26 as they were reviews; 18 as they were not in English. The remaining articles (*n* = 98) were selected for the title and abstract screening to evaluate whether they meet the PECO criteria. In all, 15 papers were excluded as a duplicate. We assessed 79 records for eligibility. Among these, 31 were excluded because they were off-topic and did not respond to PECO. The included studies have been published between 1990 and 2024. The 3 included studies were prospective, case-control, and cross sectional. All these studies compared the prevalence of bruxism between subjects exposed to epilepsy and those not. Tables 2 and 3 summarise the main characteristics of all the studies included in the present systematic review (as reported in the paragraph data extraction).

**Table 2:** Summary of the results.

Study	Population	Key findings	Significance
**Bisulli et al.**	33 epilepsy 31 control	- Higher frequency of arousal disorders (e.g. sleepwalking, sleep terrors) in NFLE families. Suggests a genetic or intrinsic link between NFLE and parasomnias.	Indicates a potential genetic predisposition or shared pathophysiological mechanisms between NFLE and arousal parasomnias.
**Khachatryan et al.**	175 epilepsy 130 control	- Increased prevalence of sleep bruxism and RLS in epilepsy patients. - Association of sleep disorders with seizure control and AED therapy.	Highlights the need for comprehensive sleep assessments in epilepsy management and the complex interplay between epilepsy, AEDs, and sleep disorders.
**Khatami et al.**	100 epilepsy 90 control	- Confirmed the high prevalence of arousal disorders among NFLE probands and relatives. - Explored the genetic background and neurophysiological mechanisms linking NFLE with sleep-related movement disorders.	Provides evidence supporting the hypothesis of a common pathogenic basis between NFLE and certain parasomnias, emphasising the role of genetic factors and abnormal arousal mechanisms.

NFLE: nocturnal frontal lobe epilepsy; AED: antiepileptic drug; RSL: Restless legs syndrome.

**Table 3:** Principal elements of the studies which formed part of the present systematic analysis.

Author	Study design	Population characteristic	Tools to evaluate	Results
Khatami et al.	Prospective observational study	The population characteristics include a mix of focal and generalised epilepsy, a mean age of around 45 years, a higher proportion of males in the epilepsy group, a significantly higher BMI in the epilepsy group, and the inclusion of healthy subjects recruited from hospital staff and their relatives.	Epworth Sleepiness Scale	bruxism (10% vs. 19%, *p* = 0.07)
Bisulli et al.	Case-control family study, non-randomised, non-blinded, observational, single-site, retrospective	The population characteristics include NFLE probands recruited from the Epilepsy and Sleep Centres of the Department of Neurological Sciences, University of Bologna, and a total of 458 individuals consisting of NFLE probands, relatives of probands, controls, and control relatives. The demographic features of NFLE probands and controls were similar, with the relatives of NFLE probands being approximately 4 years older than the relatives of controls and including fewer unmarried subjects.	ICSD-R criteria	37.5% Epilepsy vs 10 *p*-value: 0.017
Khachatryan et al.	Cross-sectional design	- The study includes 175 patients with epilepsy (PWE) with an age range of 18–71 years and a mean age of 35.4 years, with 47.4% being female. – The control group consists of 130 individuals with an age range of 18–72 years and a mean age of 33.6 years, with 47.7% being female. – The study specifically focuses on adult PWE aged 18 years and above who were admitted to a tertiary epilepsy center for assessment of their seizure disorders. – The control group participants were randomly recruited and had no relationship with the epilepsy group. They were adults aged 18 and above, fluent in Armenian language, with normal communication ability, and had no history of epilepsy or loss of consciousness episodes. They also had no known severe medical or neuropsychiatric disorders.	International Classification of Sleep Disorders, Third Edition	23.7% for Epilepsy VS 5.4 for control

OR: odds ratio; CI: confidence interval; NFLE: nocturnal frontal lobe epilepsy; ICSD: International Classification of Sleep Disorders-Revised.

### Main findings

The included subjects in this review were 559. Among them, 308 were exposed to epilepsy, whereas the remaining 251 were not. Symptoms and evaluation of bruxism were done by different scales from all 3 studies considered.

The number and demographic characteristics of the enrolled patients in the 3 studies are presented in Tables 2 and 3.

Khachatryan’s study aims to assess whether sleep disorders are more frequent in patients with epilepsy than in the control group. A total of 175 epilepsy patients and 130 controls were enrolled. The patients were followed up at a clinic, after which they were asked to fill out a questionnaire containing all possible sleep disorders. Results regarding night bruxism showed that patients with epilepsy had a higher incidence of night bruxism (23.7% versus 5.4%, *p* < 0.05) [27].

The purpose of Khatami’s study is to assess the presence of sleep disorders in a group of patients with epilepsy, specifically bruxism. Sleep disorders were assessed through a clinical interview and a questionnaire. In all, 100 patients with epilepsy and 90 healthy patients were enrolled. The study showed, once data regarding bruxism were extrapolated, that 10% of epilepsy patients suffered from bruxism, while 19% of healthy patients suffered from bruxism [28].

Bisulli’s study evaluated a group of patients with frontal lobe epilepsy. The study is structured as a retrospective observational study. It also considered relatives of frontal lobe epilepsy patients in addition to the control group precisely to assess whether there was a genetic correlation regarding the frequency of sleep disorders. A total of 33 epilepsy patients, 200 relatives, 31 controls and 191 relatives of the controls were considered. Patients were examined by telephone or questionnaire. Comparison between patients with frontal epilepsy and control regarding nocturnal bruxism showed that 12 had bruxism in the study group and 3 in the control group [29].

### Meta-analysis

**Figure 2:**
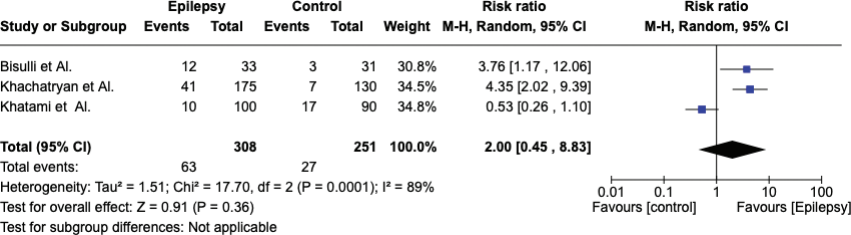
Forest plot.

The meta-analysis was conducted by random model effect because of the high heterogeneity (*I*^2^ = 89%) among the three included studies that compared the prevalence of bruxism in epilepsy patients and control subjects. The outcome chosen to compare the three studies was the prevalence of bruxism. The overall effect, reported in the forest plot (Figure 2), revealed that subjects exposed to epilepsy had a higher risk to develop bruxism than controls not exposed to caffeine (RR [relative risk] 2.00; 95% CI [confidence interval]: 0.45– 8.83; *Z* = 0.91; *p* = 0.36), but not statistically significative.

### Quality assessment and risk of bias

**Figure 3:**
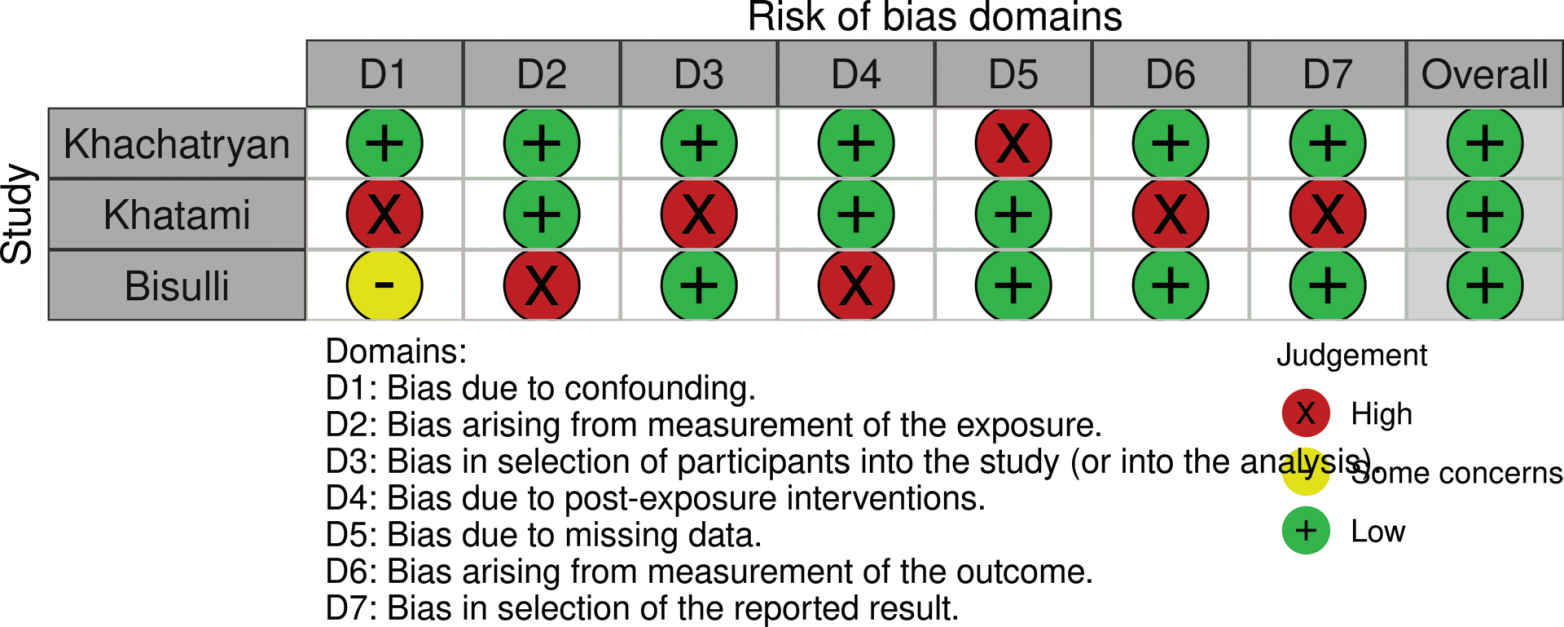
Bias assessment.

The bias was assessed with the ROBINS-E tool and is reported in Figure 3. All the studies show a low risk of bias. With regard to the bias due to confounding, only the Khachatryan’s study shows a low risk of bias. The measurement bias is low in almost all studies. Two studies show a low risk of bias due to participant selection. Also, two studies show a low bias due to measurements, although they were carried out by means of questionnaires. The bias due to the presentation of results is negligible and low. We can say as a final judgement that the overall bias of the three studies is low grade Table 4 shows a high level of evidence. Despite the heterogeneity of the studies, the results of the meta-analysis are evident and therefore leave no doubt about the evidence. However, the small sample and heterogeneity of the methods used affected the result by showing a lack of association between epilepsy and bruxism

**Table 4:** Level of evidence.

Certainty assessment							No of patients		Effect		Certainty	Importance
No of studies	Study design	Risk of bias	Inconsistency	Indirectness	Imprecision	Other considerations	Schizophrenic	Healthy	Relative (95% CI)	Absolute (95% CI)		
**New outcome**												
3	Non-randomised studies	Not serious	Not serious	Not serious	Not serious	None	308 Cases 251 controls 63/308 exposed 27/251 unexposed		Not estimable	-	⊕⊕⊕⊕ High	IMPORTANT
							-	0.0%				

CI: confidence interval.

## Discussion

Bruxism and epilepsy are two distinct neurological conditions that have garnered considerable attention in the medical field due to their impact on patients’ quality of life and potential underlying connections. Bruxism, characterised by involuntary teeth grinding and clenching, and epilepsy, a chronic neurological disorder involving recurrent seizures, have been studied independently for decades [30]. Recently, researchers have begun investigating a potential association between these two conditions. This scientific discussion aims to explore the existing evidence and hypotheses linking bruxism and epilepsy, and sheds light on their possible interrelationship.

Bruxism and its Mechanisms: Bruxism involves rhythmic jaw muscle activity that results in teeth grinding and clenching during sleep or wakefulness. Sleep bruxism, specifically, is considered a sleep-related movement disorder and is thought to involve the central nervous system. The exact pathophysiological mechanisms leading to bruxism remain unclear. However, it is commonly associated with increased levels of stress, anxiety, and certain medications, such as psychostimulants. It has been proposed that disturbances in the central dopaminergic pathways might also play a role in the development of bruxism.

The discussion synthesises findings from three comprehensive studies on sleep-related disorders in epilepsy patients, highlighting the intricate relationship between epilepsy, particularly nocturnal frontal lobe epilepsy (NFLE), and various sleep disturbances. These studies collectively reveal a heightened prevalence of sleep disorders among epilepsy patients compared to control groups, underscoring the multifaceted impact of epilepsy on sleep quality and the occurrence of parasomnias [31].

A pivotal observation across the studies is the significant presence of arousal disorders and sleep-related movement disorders in individuals with epilepsy. This suggests an underlying common pathophysiological mechanism linking epilepsy, especially NFLE, to disturbances in sleep architecture. The studies report a range of sleep disturbances, including insomnia, unrefreshing sleep, poor sleep hygiene, excessive daytime sleepiness, sleep paralysis, snoring, breathing pauses during sleep, restless legs syndrome, and sleep bruxism. Notably, the incidence of restless legs syndrome and sleep bruxism was markedly higher in epilepsy patients, indicating a potential shared neural pathway or the impact of chronic neurological conditions on sleep-related motor functions.

Furthermore, the studies delve into the implications of AED therapy on sleep disorders, presenting mixed findings. While some correlations between AED therapy and specific sleep disorders were observed, such as a higher prevalence of sleep attacks in patients receiving pharmacotherapy, the overall impact of AEDs on sleep architecture remains complex and warrants further investigation. This highlights the need for careful consideration of AEDs’ effects on sleep in the management of epilepsy.

The comparative analysis within the epilepsy group, examining focal versus generalised epilepsies and the influence of AED therapy, did not reveal statistically significant differences in sleep disorders prevalence. This suggests that the type of epilepsy and AED therapy may not be primary determinants of sleep disturbances, pointing instead to the inherent association between epilepsy and altered sleep patterns [32].

Epilepsy is characterised by abnormal and excessive electrical activity in the brain, leading to recurrent seizures. Seizures can vary in their manifestation and severity, depending on the affected brain regions. While the exact aetiology of epilepsy is multifactorial and often idiopathic, it can be caused by various factors, including genetic predisposition, brain injury, infections, and developmental abnormalities. Changes in ion channels and neurotransmitter imbalances are considered important contributors to the development of epilepsy.

The link between bruxism and epilepsy has emerged from several clinical studies and case reports, presenting intriguing correlations. Some individuals with epilepsy have been reported to display bruxism episodes, both during the daytime and sleep. Furthermore, the co-occurrence of bruxism and epilepsy in patients has sparked interest in the possible underlying mechanisms that might connect these seemingly unrelated conditions.

Both bruxism and epilepsy involve dysregulation of the central nervous system, and it is possible that they may share some common neural pathways or brain regions involved in their manifestation. Abnormal activity in regions like the basal ganglia and the cortex has been implicated in both bruxism and epilepsy, suggesting a potential overlapping pathway.

Certain antiepileptic medications used to manage epilepsy, such as phenytoin, valproate, and lamotrigine, have been associated with side effects like bruxism. This raises the question of whether these medications may directly contribute to the development of bruxism in patients with epilepsy or exacerbate pre-existing bruxism [33–37].

Patients with epilepsy often face significant psychological stress due to the unpredictable nature of seizures and potential limitations in daily life. Similarly, psychological stress and anxiety have been recognised as potential triggers for bruxism. It is possible that the psychological burden of epilepsy may contribute to the development or worsening of bruxism in some individuals.

While there is emerging evidence suggesting a potential association between bruxism and epilepsy, the exact nature of this relationship remains elusive and requires further investigation. Shared neural pathways, the influence of antiepileptic medications, and psychological factors are among the key areas that warrant deeper exploration. Understanding the possible links between bruxism and epilepsy could provide valuable insights into the underlying mechanisms of both conditions and potentially open avenues for improved management strategies and patient care. As research progresses, clinicians and researchers should be vigilant in considering the presence of both conditions in their patients and remain open to the complexities of this intriguing association.

The limitations of our review largely stem from a small sample size, and the heterogeneity among the included studies proved so high that we were unable to gain statistically significant results. In total, only three studies were incorporated into the analysis, leaving us with a very low availability of data and very highly limited generalisability of the findings. The heterogeneity also was affected by the variation in measuring bruxism methods, for instance in different scales and approaches to diagnostic tests, *I*² = 89%. As such, the results cannot be consistent. Measurements were self-reported in some studies, which introduced bias to the measurement and bruxism prevalence estimates. Of course, high levels of evidence were also shown in the GRADE assessment for this analysis. That said, the heterogeneity of study designs and the differences between the populations surveyed limited the complexity of the interpretation of results. These factors somewhat limit the ability to draw firm conclusions about the association between epilepsy and bruxism.

Given the complexity of the potential association between bruxism and epilepsy, further research is warranted to establish a more concrete link and understand the underlying mechanisms. Following are some potential avenues for future research:

– Long-term, prospective studies that follow individuals with epilepsy and monitor the development of bruxism over time would be valuable in determining whether there is a causal relationship between the two conditions or if they are merely co-occurring due to shared risk factors.
– Advanced neuroimaging techniques, such as functional magnetic resonance imaging (fMRI) and positron emission tomography (PET), could be employed to investigate brain activity patterns in patients with both bruxism and epilepsy. This would provide insights into potential common brain regions or neural pathways involved in both conditions.
– Experimental studies could investigate the effects of antiepileptic medications on bruxism development or exacerbation in animal models. These studies could provide valuable information about the role of specific drugs in the interplay between bruxism and epilepsy [38–40].

Genetic research may shed light on whether there are shared genetic factors predisposing individuals to both bruxism and epilepsy. Genome-wide association studies (GWAS) and familial investigations could identify specific genetic variants that might be relevant to both conditions.

*Intervention Studies*: Clinical trials could be conducted to assess the impact of epilepsy management strategies on bruxism occurrence and severity. For example, investigating whether modifying AED regimens or introducing stress management techniques reduces bruxism episodes in patients with epilepsy.

Healthcare providers treating patients with epilepsy should be aware of the potential co-occurrence of bruxism and consider its impact on patients’ oral health and overall well-being. Routine dental examinations could help identify bruxism in epilepsy patients and enable early intervention.

Patients diagnosed with epilepsy should be educated about the possible association with bruxism, its potential impact on oral health, and strategies for managing stress and anxiety, which could help reduce bruxism episodes.

Collaborative efforts between neurologists, dentists, and sleep specialists would enhance the understanding of the relationship between bruxism and epilepsy and lead to more comprehensive patient care.

*Individualised Treatment*: Considering the potential influence of antiepileptic medications on bruxism, healthcare providers should tailor treatment plans for each patient, weighing the benefits of seizure control against the risk of exacerbating bruxism.

## Conclusion

Bruxism and epilepsy represent intriguing neurological conditions with potential interconnections that necessitate further exploration. Although evidence supporting a definitive link is still limited, the shared neural pathways, the influence of antiepileptic medications, and psychological factors suggest a possible association between the two conditions. As research progresses, clinicians and researchers should remain vigilant and consider the presence of both conditions in their patients to provide optimal care and improve the quality of life for individuals affected by bruxism and epilepsy. Collaboration among various medical disciplines will be instrumental in elucidating the relationship between these two complex conditions and advancing patient care in the future.

## Data Availability

The data will be available on reasonable request from the corresponding author.
